# Habitat Type and Disturbance Intensity Are Associated With Invasive Plant Community Structure Across an Urbanizing Subtropical Island

**DOI:** 10.1002/ece3.74522

**Published:** 2026-10-20

**Authors:** Sha Wang, Ming Li, Yanan Guo, Jun Li, Ruijiang Wang

**Affiliations:** ^1^ State Key Laboratory of Plant Diversity and Specialty Crops, South China Botanical Garden Chinese Academy of Sciences Guangzhou Guangdong China; ^2^ Key Laboratory of National Forestry and Grassland Administration on Plant Conservation and Utilization in Southern China, South China Botanical Garden Chinese Academy of Sciences Guangzhou Guangdong China; ^3^ College of Life Sciences University of Chinese Academy of Sciences Beijing China; ^4^ Urban Planning and Construction Bureau of the Guangdong‐Macao In‐Depth Cooperation Zone in Hengqin Zhuhai Guangdong China

**Keywords:** habitat occupancy, habitat type, invasive intensity, plant invasion, seasonality, urban ecology

## Abstract

Urbanization promotes biological invasions, yet the relative importance of spatial versus temporal dynamics in subtropical urban ecosystems remains unclear. We investigated invasive plant communities across six habitat types in Hengqin, South China, to evaluate factors influencing their distribution across habitats and seasons. Using nonmetric multidimensional scaling (NMDS) and zero–one inflated Beta (ZOIB) models, we found that habitat type and disturbance intensity were strongly associated with invasive species distribution, explaining 58.9% of the community composition (*R* ^2^ = 0.589). Seasonal variation showed limited influence on community composition (*R* ^2^ = 0.012, *p* = 1.000), but the ZOIB model revealed a significant habitat × season interaction ($\chi_{15}^{2}$ = 30.32, *p* = 0.011), indicating that seasonal effects were habitat‐dependent. Invasive species exhibited significantly broader occupancy breadth than native species (Shannon index: 3.00 vs. 2.45, *p* = 0.024), consistent with the generalist hypothesis. Greater spatial co‐occurrence among dominant invasive species (median = 0.203) compared to native species suggests that these plants share similar habitat preferences and disturbance tolerance, enabling co‐occurrence in high‐stress environments. The weak overall seasonal signal contrasts with phenology‐based models typical of temperate zones, although seasonal effects were evident in certain habitat types (e.g., forest edge, park, and wetland declined from spring to summer). This relative stability may reflect subtropical climate and the ability of dominant invasive species to maintain year‐round growth. These findings suggest that management should prioritize high‐risk habitats such as roadside and wasteland, while also considering seasonal timing in habitats with marked fluctuations.

## Graphical abstract

This study investigated invasive plant communities across a disturbance gradient in Hengqin, China, to evaluate how habitat characteristics and seasonal variation influence community assembly. The findings indicate that habitat type and its associated disturbance regimes are the primary predictors of invasive distribution, accounting for 58.9% of community variation, while seasonality had minimal impact. Invasive plants exhibited broader ecological niches compared to native plants, allowing them to thrive in high‐disturbance environments. The lack of significant seasonal variation may be due to the mild subtropical climate, suggesting management efforts should prioritize the restoration of high‐disturbance habitats over seasonal timing.

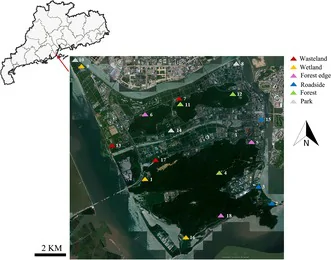

## 1 Introduction

Rapid urbanization is a primary factor leading to habitat loss and fragmentation, creating novel environments where plant invasions pose a significant threat to native biodiversity (Hobbs and Huenneke 1992). The success of alien species in these modified landscapes is often examined through concepts related to resource use and environmental tolerance. These concepts suggest that invasive species thrive by exploiting available resources. Traditionally, invasion success is viewed as a function of habitat filtering (spatial variation) and seasonal dynamics (temporal variation) (Odling‐Smee et al. 2003; Guisan et al. 2014). However, the relative importance of these two factors remains debated, particularly in subtropical urban environments. While seasonal phenology is a critical factor in temperate zones (Wolkovich and Cleland 2011), it is unclear whether invasion risk in subtropical cities follows a seasonal phenological pulse or is more consistently associated with habitat disturbance. Analyzing the spatiotemporal patterns and habitat occupancy characteristics of invasive species is therefore essential for understanding their ecological strategies and informing management (Petitpierre et al. 2016; Early et al. 2016).

Compared with traditional broad‐scale surveys, finer‐scale research on the interplay between habitat types and temporal dynamics is increasingly needed. Although multiyear studies exist (Meiners et al. 2002; Martin et al. 2009; Kuebbing and Nuñez 2015), few have simultaneously quantified how habitat‐specific environmental filters (i.e., the abiotic and biotic conditions associated with different land uses) interact with seasonal changes in plant cover and composition within rapidly urbanizing contexts. Specifically, the high heterogeneity of urban landscapes, ranging from preserved forests to highly disturbed wastelands, provides a unique opportunity to test these interactions (Seto et al. 2011). In this context, the Guangdong‐Macao In‐Depth Cooperation Zone in Hengqin, a rapidly urbanizing island over the past 15 years, serve as an ideal model system. It is characterized by intensive construction, fragmented habitats, and a subtropical climate, and exhibits elevated invasion risks and diverse habitat conditions (Kueffer et al. 2010; Gaertner et al. 2017).

Since 2009, extensive development in Hengqin has resulted in a pronounced gradient of anthropogenic disturbance (Ye et al. 2012). While significant progress has been made in understanding invasion patterns globally (Pyšek et al. 2020), local research often focuses on static species lists or physiological traits (Wu et al. 2010), lacking a systematic, community‐level perspective on spatiotemporal dynamics. Understanding how invasive communities assemble across different habitat types and how they spatially co‐occur with native species remains a key knowledge gap (MacDougall et al. 2009; Gallien and Carboni 2017).

To address these knowledge gaps, we conducted a multi‐season investigation of invasive plant communities across six habitat types in Hengqin Island. We aimed to answer three key questions: (1) how does the cover of invasive species vary across habitats with different disturbance levels, and is this variation consistent across seasons? (2) how does the community composition of invasive species differ among habitat types? (3) what is the degree of spatial co‐occurrence between dominant invasive species and native species? By integrating community ecology with occupancy analysis, this study aims to identify factors associated with invasion in urbanized landscapes and provide a scientific basis for management strategies.

## 2 Materials and Methods

### 2.1 Study Area and Sampling Design

Hengqin is situated in southern Zhuhai City, Guangdong Province, China, and is characterized by a South Subtropical Monsoon climate with a mean annual temperature of 22°C*–*23°C and annual precipitation of approximately 2015.9 mm. Following the development of the Guangdong‐Macao In‐Depth Cooperation Zone, the region has undergone rapid urbanization, consequently forming a complex spatial pattern where natural ecosystems coexist with environments that are subject to high‐intensity human disturbance. Hengqin island has a land area of approximately 106.46 km^2^, and its relatively compact spatial scale reduces broad‐scale climatic and geological variation, allowing us to focus on habitat‐level and disturbance‐related effects on invasive plant communities.

To capture the main habitat types in Hengqin, we first conducted a reconnaissance survey to identify the major habitat types present in the study area. Based on this preliminary assessment, we classified the landscape into six representative habitat types: forest, park green space, roadside, wetland, forest edge, and wasteland. Within each habitat type, we established three fixed monitoring plots (totaling 18 plots) using a random sampling design based on GPS coordinates. Specifically, for each habitat type, we generated random GPS points within the distribution range of that habitat using the random point generation function in OvitalMap (a widely used mobile GIS application for field surveys). These coordinates were then located in the field using the navigation function of OvitalMap on a mobile device. If a generated point fell in an inaccessible area (e.g., private property, steep terrain) or was unrepresentative of the habitat (e.g., a road within a forest stratum), it was discarded and a new random point was generated. To mitigate potential spatial autocorrelation, plots within the same habitat type were separated by at least 500 m. All plots were established between March 2023 and December 2024. The localities of these plots are shown in Figure 1.

**FIGURE 1:**
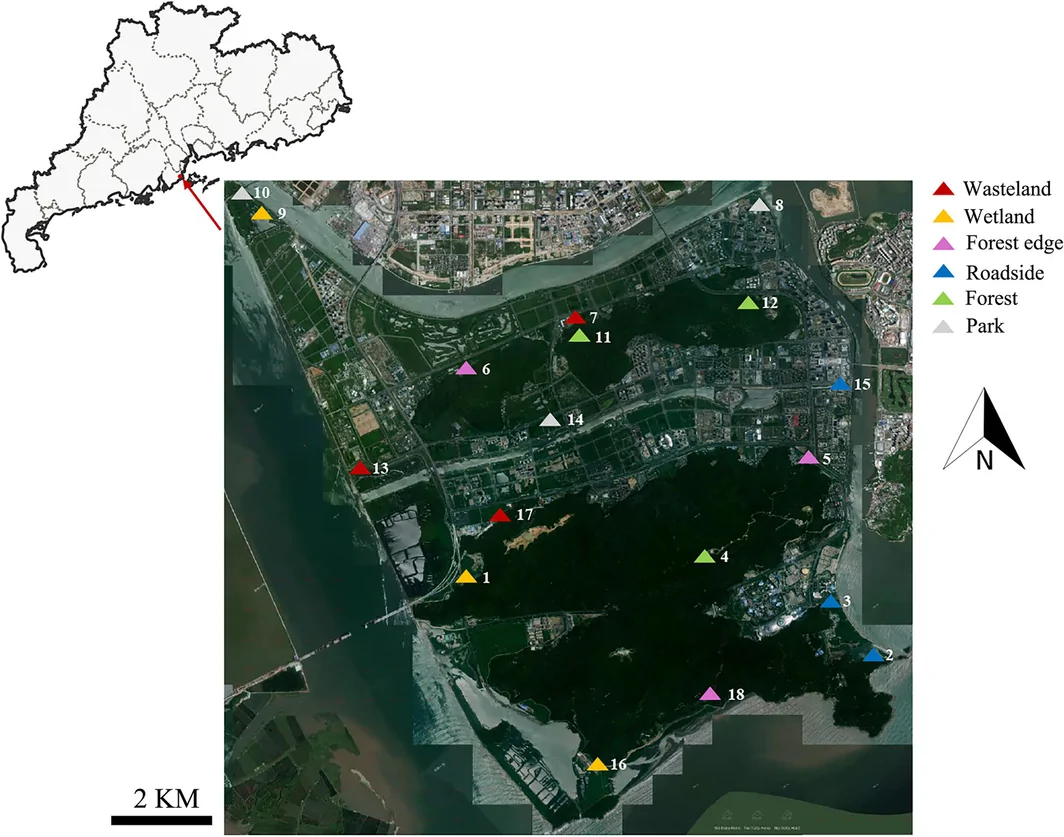
Location of the 18 monitoring plots for invasive alien plants in Hengqin, Zhuhai City, Guangdong Province, China. The base satellite image is sourced from OvitalMap (Google Earth imagery, accessed 2024). Inset map shows the location of Hengqin within Guangdong Province.

Six habitat types were classified and defined as follows: (1) forest: subtropical secondary forests characterized by closed canopies, multilayered vegetation composition, and minimal recent anthropogenic modification (e.g., no logging or trail construction within the plot); (2) wetland: typical wetland areas, including both freshwater ponds and estuarine mudflats subject to tidal influence, characterized by periodic flooding and hydric soils; (3) park: managed urban green spaces characterized by mowed lawns (turfgrass), with sampled plots located in open lawn areas dominated by turfgrass species, with occasional scattered trees or shrubs but no continuous tree canopy; (4) roadside: linear zones along major transportation arteries, affected by fragmented vegetation, soil compaction, traffic emissions, and edge effects; (5) forest edge: ecotonal habitats at the transition between interior forests and adjacent open areas (e.g., managed lawns, abandoned fields, or roads), reflecting mixed environmental influences from both forest and open‐land ecosystems; (6) wasteland: abandoned lands (e.g., former construction sites or fallow fields) undergoing early natural succession, typically dominated by ruderal and pioneer species.

Although each habitat type contained only three independent plots (18 plots in total), each plot covered 400 m^2^ (20 m × 20 m), a standard scale for subtropical forest community surveys in China (Fang et al. 2009) and was large enough to capture the major species composition and spatial variation within each habitat. All vascular plant species (trees, shrubs, and herbs) were recorded in each plot, covering multiple vegetation layers. Combined with the 2‐year seasonal sampling (eight surveys), this design yielded 144 observational records (18 plots × eight seasons), providing sufficient degrees of freedom for the mixed‐effects models to estimate seasonal effects and plot‐level variation.

### 2.2 Vegetation Survey

All plots were uniformly 20 m × 20 m (400 m^2^), a standard scale for vegetation surveys in subtropical China (Fang et al. 2009), which adequately captures community composition in forests, forest edges, and wetlands. For habitats with lower spatial heterogeneity (e.g., park lawns) or linear configurations (e.g., roadsides), we acknowledge that 400 m^2^ may not be fully representative; however, we retained this uniform plot size for two reasons: (1) to maintain consistency in plot area across habitats, avoiding area effects as a confounding factor in comparisons; (2) to facilitate comparability with other urban invasion studies. For roadside plots, we selected continuous areas at least 20 m along and 20 m perpendicular to the road to achieve the 400 m^2^ area while excluding road pavement. All plots were confirmed to meet these criteria prior to analysis. For species recording, each 20 m × 20 m plot was subdivided into a contiguous grid of sixteen 5 m × 5 m subplots, within which all vascular plant species were identified and their coverage estimated.

We conducted vegetation surveys in each 20 m × 20 m plot seasonally between March 2023 and December 2024, covering spring, summer, autumn, and winter (eight surveys over 2 years). In each survey, all vascular plant species (trees, shrubs, and herbs) were identified and recorded. For each species in each plot, we measured the following five parameters: (1) abundance: number of individuals or clumps. For clonal or densely growing species, abundance was estimated as the number of distinct clumps or tillers; (2) height: for each species, height (cm) was measured for 10 individuals. To minimize observer bias and ensure reproducibility, individuals were selected randomly across the spatial extent of the plot (rather than clustered in one area). For species with fewer than 10 individuals, all present individuals were measured. The average height was calculated to represent the species' vertical structure within the community; (3) species cover: canopy projection cover (%), estimated visually for each species. To improve accuracy in large plots, observers walked systematically through the entire plot along a *Z*‐shaped transect and integrated observations from at least four directions before assigning a cover value. The final cover value for each species was determined by synthesizing observations from multiple positions along the *Z*‐shaped transect, rather than from a single vantage point, to minimize edge effects and local overestimation. Estimates were made with reference to a standard cover class diagram to minimize subjectivity; (4) total vegetation cover: the total percentage of the plot area covered by the vertical projection of all vascular plant vegetation combined (overlapping canopies counted once). Invasive plant cover was estimated as the combined cover of all invasive alien species present in a plot, accounting for canopy overlap, yielding a continuous proportional value (0%–100%) for each plot. Invasive species were classified following the Checklist of Chinese invasive species (Ma and Li 2018); (5) quality control: all surveys were conducted by the same two well‐trained observers throughout the study period. Before each sampling season, the two observers conducted a calibration session in representative plots, independently estimating cover and height for selected species, then comparing and discussing discrepancies until consensus was reached. Calibration sessions were considered successful when the absolute difference between the two observers' cover estimates for the same species fell below 5% for ≥ 90% of the species assessed. If this criterion was not met, additional rounds of training were conducted until consistency was achieved. Cover estimates were periodically cross‐checked between observers during the field campaign.

### 2.3 Data Analysis

All statistical analyses and graphical visualizations were performed using R version 4.3.1 (R Core Team 2023).

#### 2.3.1 Quantification of Anthropogenic Disturbance

To quantify the level of anthropogenic disturbance across sampling sites, we recorded three indicators for each plot: (1) distance to nearest road (m): measured using GPS coordinates and satellite imagery in OvitalMap; (2) trampling intensity: assessed on a 1–5 scale based on visible footpaths, soil compaction, and vegetation damage, with explicit criteria for each level: 1 = no visible footpaths or trampling; 2 = occasional footprints but no continuous trail; 3 = distinct footpaths covering < 30% of the plot; 4 = well‐defined trails covering 30%–60% of the plot with localized soil compaction; 5 = extensive trail network or bare soil covering > 60% of the plot with severe compaction; (3) intensity of anthropogenic traces: scored on a 1–5 scale based on the abundance of anthropogenic traces (litter, pruning/cutting marks, vehicle tracks, construction debris) recorded during a standardized 30‐min walk‐through survey: 1 = no visible traces; 2 = occasional traces (< 5 items or marks); 3 = moderate traces (5–20 items); 4 = abundant traces (20–50 items); 5 = highly abundant traces (> 50 items or continuous evidence of intensive use).

All disturbance scores were recorded independently by two well‐trained observers during plot establishment. Interobserver agreement was assessed using Cohen's Kappa, with values of κ = 0.556 for trampling intensity and κ = 0.874 for intensity of anthropogenic traces, indicating “moderate” and “almost perfect” agreement, respectively (Landis and Koch 1977). After independent scoring, the two observers compared their results and discussed any discrepancies to reach a consensus on the final scores.

These three variables were combined into a single disturbance index using principal component analysis (PCA). All variables were scaled to zero mean and unit variance prior to analysis. The first principal component (PC1), which explained 71.7% of the total variance, was used as the continuous disturbance index in subsequent analyses (see Figure S1 and Table S1 for detailed results). This approach allowed us to incorporate a quantitative measure of disturbance alongside the categorical habitat types.

#### 2.3.2 Analysis of Community Composition

To investigate how invasive plant community composition varied across habitats, we used a species composition matrix comprising only the 58 invasive species recorded in the study, with species cover as the response variable.

We performed a permutational multivariate analysis of variance (PERMANOVA) based on Bray‐Curtis dissimilarities (Anderson 2001; McArdle and Anderson 2001) including habitat type, season, and their interaction as explanatory variables. This allowed us to test for both habitat effects and seasonal variation in community composition. Nonmetric Multidimensional Scaling (NMDS) was used to visualize community dissimilarity patterns, and the stress value was reported to evaluate the goodness of fit (Kruskal 1964; Clarke and Warwick 2001).

Prior to interpreting the PERMANOVA results, we tested the assumption of homogeneous multivariate dispersions among habitats using PERMDISP (betadisper function in the *vegan* package; Anderson 2006). This test examines whether significant PERMANOVA results could arise from differences in within‐group variance rather than shifts in group centroids.

To quantify the relative contributions of habitat type, season, and disturbance index (PC1) to variation in invasive community composition, we performed distance‐based redundancy analysis (dbRDA; capscale function in the *vegan* package) using Bray‐Curtis dissimilarities as input. dbRDA is a constrained ordination method that is more appropriate for community data with high zero‐inflation and potentially unimodal species responses than linear methods such as RDA (Legendre and Anderson 1999). Habitat type was included as a categorical variable (with forest as the reference level), and season was included as a categorical variable (with autumn as the reference level). Disturbance_Index (PC1) was included as a continuous covariate. The significance of the overall model and of each explanatory variable was assessed using permutation tests (999 permutations).

#### 2.3.3 Analysis of Species Occurrence Patterns

To evaluate the spatial distribution strategies of invasive and native plants, we calculated habitat occupancy breadth and spatial co‐occurrence. Following the recommendation to prioritize standard metrics, we primarily used Levins' index to calculate habitat occupancy breadth ($B_{L}$; Levins 1968), and calculated Shannon's index for occupancy breadth ($B_{s}$) as a sensitivity analysis to ensure robustness.

The Levins index is calculated as:

$$B_{L}=1/\sum \left(P_{\mathrm{ij}}^{2}\right)$$

The Shannon index is calculated as:

$$B_{S}=-\sum \left(P_{\mathrm{ij}}\ln P_{\mathrm{ij}}\right)$$

In the formula, $P_{\mathrm{ij}}$ represents the proportion of the cover of species i in spatiotemporal sampling unit j relative to its total cover across all units. In this study, spatiotemporal sampling units are defined as the 72 sampling units (18 plots × four seasons). To capture generalized seasonal patterns and mitigate interannual weather anomalies, species cover data from the same seasons across the two sampling years (2023 and 2024) were averaged for each plot. This resulted in a robust dataset of 72 spatiotemporal sampling units representing heterogeneity across the study area. We acknowledge that this approach treats each plot‐by‐season combination as a discrete unit for occupancy assessment, rather than directly measuring specific environmental resource axes (e.g., light, moisture, nutrients), thus, the breadth and co‐occurrence values reported here reflect spatial distribution patterns across habitat‐season combinations, rather than direct measures of resource utilization or competitive displacement. A higher index value indicates a broader habitat occupancy breadth.

To select dominant species for occupancy analysis, we used threshold‐based criteria rather than a fixed number of species. Species were considered dominant if they occurred in at least 20% of the 72 sampling units and had a mean cover > 1.5%. To test the robustness of our results to the choice of thresholds, we conducted a sensitivity analysis by varying the occurrence threshold (15%, 20%, 25%) and cover threshold (1%, 1.5%, 2%). The set of dominant species remained largely consistent across these thresholds, and the main patterns (broader occupancy breadth and higher co‐occurrence for invasive species) were unchanged.

Based on these criteria, we selected seven invasive species and eight native species for further analysis (see Table S2). The selected invasive species were: *Bidens alba* , *Sphagneticola trilobata* , *Leucaena leucocephala* , *Mikania micrantha* , *Panicum repens* , *Lantana camara* , and *Sesbania cannabina* . The selected native species were: *Cynodon dactylon* , *Phragmites australis* , *Saccharum arundinaceum* , *Ficus concinna*, *Acacia confusa* , *Ruellia simplex* , *Apluda mutica* , and *Coccinia grandis* .

To quantify potential spatial associations between dominant invasive and native species, we calculated spatial co‐occurrence using the Pianka index ($O_{\mathrm{jk}}$; Pianka 1973). We selected Pianka's index over alternative metrics (e.g., Schoener's *d*) because it is less sensitive to zero values and is widely used in plant community ecology for assessing pairwise species co‐occurrence (Gotelli and Ellison 2004). The formula is:

$$O_{\mathrm{jk}}=\sum \left(P_{\mathrm{ij}}P_{\mathrm{ik}}\right)/\sum P_{\mathrm{ij}}^{2}\sum P_{\mathrm{ik}}^{2}$$

where $P_{\mathrm{ij}}$ and $P_{\mathrm{ik}}$ are the proportions of the cover of species j and species k in sampling unit i. Values range from 0 (no co‐occurrence) to 1 (complete co‐occurrence).

We then calculated co‐occurrence for all possible pairwise combinations among these 15 dominant species (total 105 pairs), which were categorized into three groups: (1) invasive‐native pairs (*n* = 56), (2) invasive‐invasive pairs (*n* = 21), and (3) native‐native pairs (*n* = 28). All calculations were performed in R using the *spaa* package (Zhang 2024).

#### 2.3.4 Factors Associated With Invasive Plant Cover

To investigate the effects of habitat type, season, and their interaction on invasive plant cover, we fitted zero–one inflated Beta (ZOIB) mixed models using the *glmmTMB* package in R (Brooks et al. 2017). This modeling approach was chosen for three reasons: (1) the response variable (invasive plant cover per plot) is a bounded continuous proportion (0%–100%, expressed as proportions between 0 and 1) and contained both excess zeros and excess ones; (2) the data exhibited overdispersion, making Beta distribution appropriate; and (3) the study design included repeated measurements on the same plots across seasons, requiring the inclusion of random effects.

The model was specified as follows: (1) fixed effects: habitat type (six levels), season (four levels), and their interaction; (2) random effect: plot identity, to account for repeated measurements across the eight seasonal surveys; (3) conditional model: Beta distribution with a logit link, which is appropriate for continuous proportion data bounded between 0 and 1; (4) Zero‐inflation model: a binomial distribution with a logit link, modeling the probabilities of zero inflation and one inflation separately.

Model selection was guided by Akaike Information Criterion (AIC) comparisons, and the final model was validated by inspecting residual plots using the *DHARMa* package (Hartig 2022). Significance of fixed effects was assessed using likelihood ratio tests (LRT) comparing full and reduced models. Post hoc pairwise comparisons for significant habitat × season interactions were conducted using the *emmeans* package with Tukey adjustment for multiple comparisons.

As a sensitivity analysis, we also fitted a linear mixed‐effects model with logit‐transformed proportional cover (logit transformation: log((y + ε)/(1−y + ε)), where ε = 0.01) to test the robustness of the ZOIB model results. The two models were qualitatively consistent in the direction and significance of the main effects (see Table S3).

## 3 Results and Analysis

### 3.1 Variation in Plant Community Composition

A total of 58 invasive alien plant species belonging to 18 families were recorded (see Table S4). PERMANOVA analysis indicated that community composition differed significantly across the six habitat types (PERMANOVA: pseudo‐*F*_5,12_ = 8.61, *R* ^2^ = 0.589, *p* = 0.001; Figure 2a). The NMDS ordination (stress = 0.174) showed that invasive plant communities were highly habitat‐specific, with all six habitat types forming distinct, nonoverlapping clusters (Figure 2a). Notably, forest, park, and roadside habitats occupied isolated regions of the ordination space, representing highly specialized species assemblages. In contrast, forest edge, wasteland, and wetland plots were positioned in closer proximity to one another, though their confidence ellipses remained largely separate, indicating a degree of compositional transition among these habitats. PERMDISP analysis revealed that multivariate dispersions did not differ significantly among habitats (*F* = 2.31, *p* = 0.054), confirming that the significant PERMANOVA result reflects differences in group centroids rather than heterogeneous within‐group variances.

**FIGURE 2:**
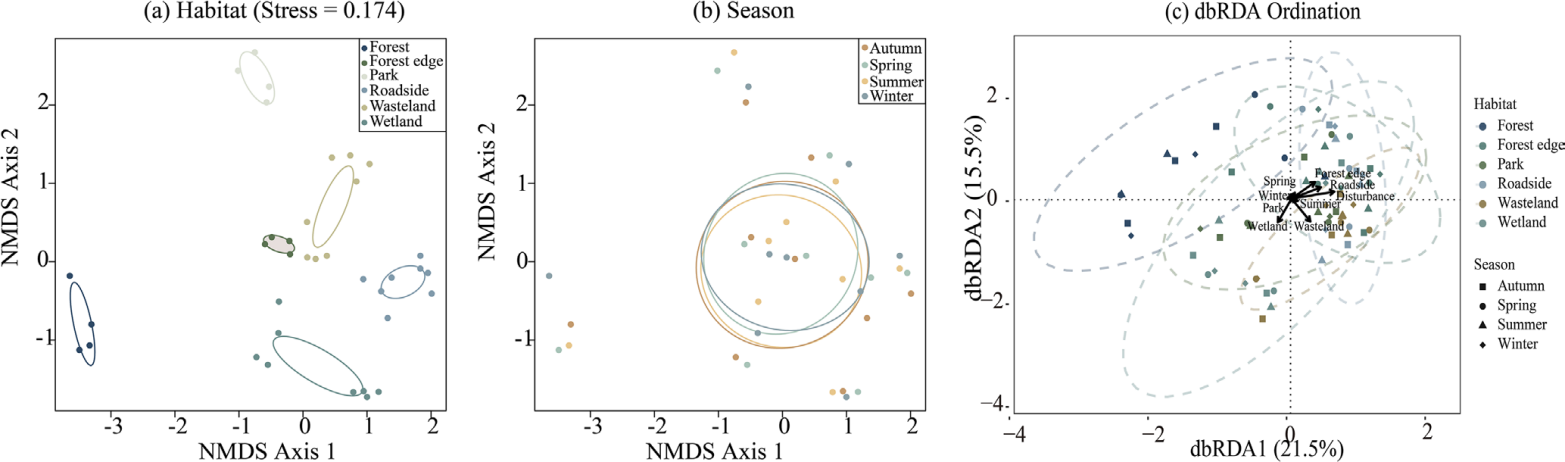
NMDS ordination of invasive plant community composition across (a) Six habitat types (stress = 0.174) and (b) four seasons. (c) dbRDA ordination illustrating the influence of habitat, season, and disturbance index on community composition. The first two axes explained 21.5% and 15.5% of the constrained variation, respectively. Marginal tests showed that habitat (*F* = 3.31, *p* = 0.001) and PC1 (*F* = 4.00, *p* = 0.001) significantly contributed to community composition, while season (*F* = 0.64, *p* = 0.955) did not. In (a) and (b), ellipses represent the 95% confidence intervals for each group.

In contrast, seasonality did not significantly influence community composition (PERMANOVA: pseudo‐*F*_3,14_ = 0.13, *R* ^2^ = 0.012, *p* = 1.000; Figure 2b). The complete intermixing of seasonal samples and the nearly concentric overlap of their confidence ellipses in the NMDS space (Figure 2b) confirms the high temporal consistency of species assemblages throughout the year.

Distance‐based redundancy analysis (dbRDA) revealed that habitat type and season together explained 37.0% of the total variation (100% of the constrained inertia; Figure 2c). The first axis (dbRDA1, 21.5%) primarily captured the divergence of Roadside habitats (positioned at the far right of the axis) from the other habitat types. The second axis (dbRDA2, 15.5%) clearly distinguished the forest plots from the remaining groups. Marginal tests indicated that habitat (*F* = 3.31, *p* = 0.001) and PC1 (*F* = 4.00, *p* = 0.001) each contributed significantly to explaining community composition, whereas Season did not retain a significant independent effect when accounting for habitat and disturbance (*F* = 0.64, *p* = 0.955). Both habitat and PC1 showed significant marginal effects, consistent with the covariation between habitat type and disturbance intensity inherent to our observational design (see Section 4).

### 3.2 Habitat Occupancy Characteristics of Invasive and Native Species

To evaluate spatial distribution patterns, we identified seven dominant invasive and eight dominant native species based on threshold criteria (see Table S5). These 15 species collectively accounted for 81.96% of the total invasive cover and 34.65% of the total native cover, ensuring broad representativeness of the community.

Overall, invasive species exhibited broader habitat occupancy compared to their native counterparts (Figure 3). For Shannon's occupancy breadth, absolute values ranged from 1.82 to 3.72 across all 15 species (Figure 3d). Statistical analysis of the standardized data (Figure 3b) confirmed that invasive species possessed significantly higher Shannon breadth (mean ± SD: 3.00 ± 0.51 in absolute terms) than native species (2.45 ± 0.28; Mann–Whitney *U* test, *p* = 0.024). A consistent, though marginally significant, trend was observed for Levins' occupancy breadth (Figure 3a), where invasive species (17.37 ± 11.53) generally scored higher than natives (9.74 ± 3.13; *p* = 0.058). Species‐level analysis (Figure 3d) highlighted *B. alba* and *L. leucocephala* as possessing the widest occupancy, although internal variation was evident among invasive species, with *P. repens* displaying a narrower occupancy than several native species.

**FIGURE 3:**
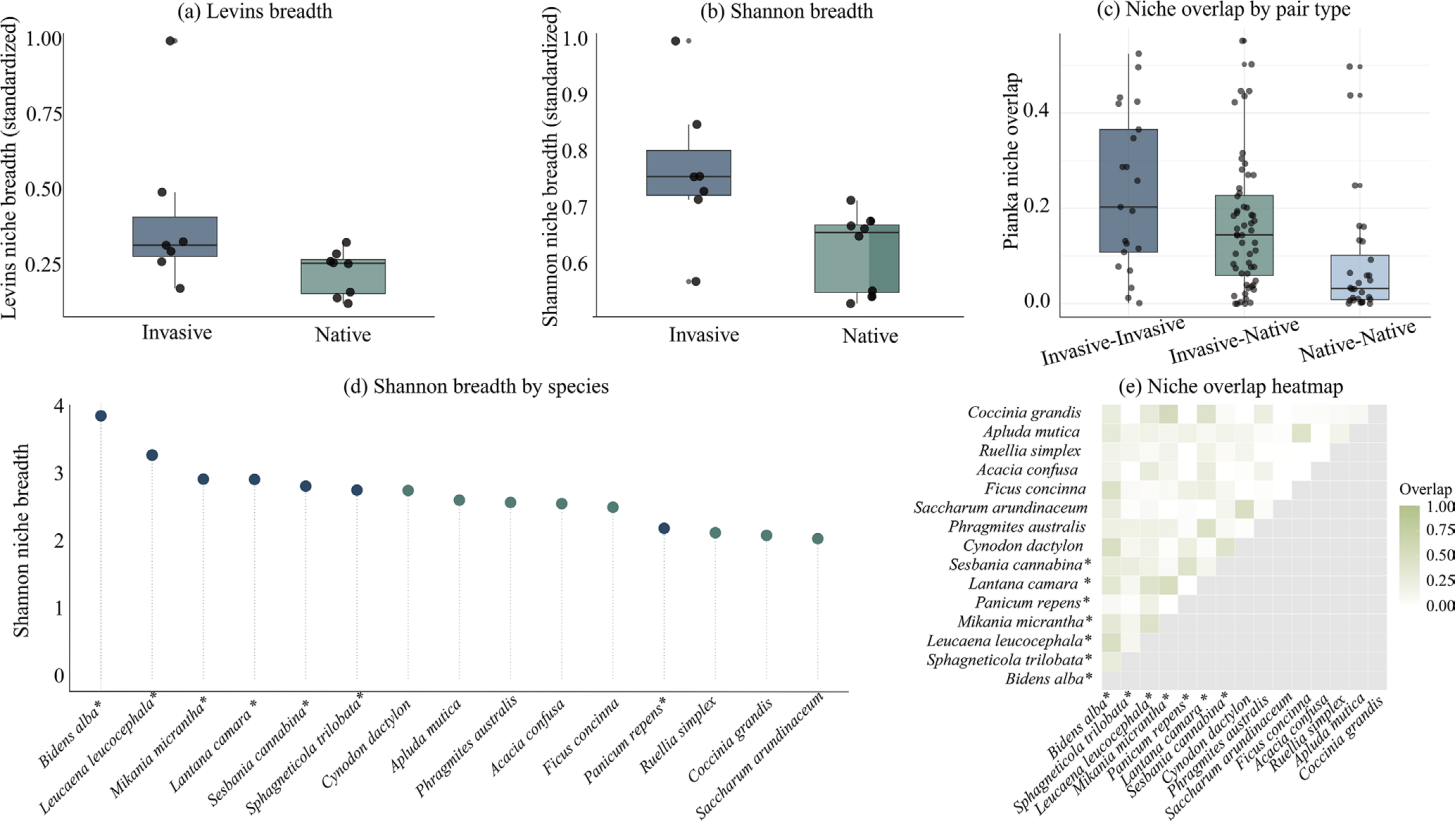
Habitat occupancy characteristics of dominant invasive and native species. (a) Standardized Levins occupancy breadth and (b) standardized Shannon occupancy breadth for invasive (*n* = 7) and native (*n* = 8) species. Boxes show median, 25th and 75th percentiles; whiskers extend to 1.5× IQR. (c) Pianka spatial co‐occurrence values for three species pair categories: Invasive–invasive (*n* = 21 pairs), invasive–native (*n* = 56 pairs), and native–native (*n* = 28 pairs). (d) Shannon occupancy breadth values for individual species (invasive species in green, native species in blue). (e) Heatmap of Pianka spatial co‐occurrence among all 15 species (seven invasive, eight native). Values range from 0 (no co‐occurrence, light colors) to 1 (complete overlap, dark green). Diagonal cells (self‐comparisons) are shaded gray. The invasive species are indicated with asterisks (*) in the species labels. To improve clarity, only the upper triangle of the pairwise matrix is shown.

Regarding spatial co‐occurrence (Pianka's index), distinct hierarchical patterns emerged among the three pair types (*p* = 0.001; Figure 3c). The highest co‐occurrence was recorded within invasive–invasive pairs (median = 0.203, IQR = 0.257), which was significantly greater than the co‐occurrence observed among native–native pairs (median = 0.032, IQR = 0.093; *p* < 0.001). Invasive–native pairs showed intermediate co‐occurrence (median = 0.144, IQR = 0.168), also significantly exceeding the native–native baseline (*p* = 0.001).

While the absolute median co‐occurrence values remained relatively low (all < 0.25), the consistently higher values within the invasive assemblage point toward greater similarity in spatial distribution patterns among invasive species compared to the more spatially segregated native species. These associations were further visualized in the heatmap (Figure 3e), which identified specific high co‐occurrence clusters involving dominant invasive species such as *B. alba* , *M. micrantha* , and *S. trilobata* . In contrast, the majority of native–native pairings occupied more distinct occupancy spaces, as indicated by the lighter regions of the heatmap.

### 3.3 Factors Associated With Invasive Plant Cover

To evaluate the factors associated with invasive plant cover, we employed a zero–one inflated Beta (ZOIB) mixed model, with plot identity as a random effect. This approach is specifically designed for bounded continuous proportion data (1%–100%, expressed as proportions 0–1) with excess zeros and ones. The model showed a good fit (AIC = −83.5; DHARMa residual diagnostics indicated no significant deviations, all *p* > 0.05) (Figure 4a).

**FIGURE 4:**
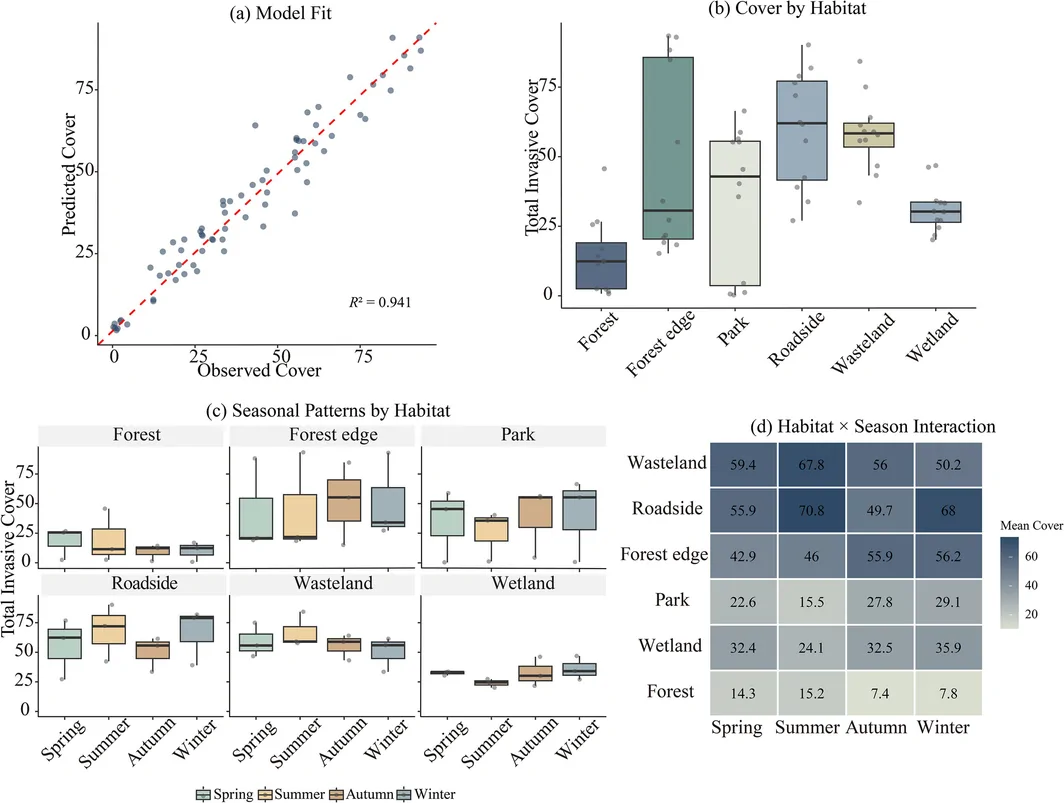
Habitat type, season, and disturbance intensity shape invasive plant cover. (a) Model fit: Observed versus predicted invasive cover from the zero–one inflated Beta (ZOIB) mixed model. The dashed line represents a 1:1 relationship. (b) Distribution of total invasive cover by habitat type. Boxes show the median, 25th, and 75th percentiles; whiskers extend to 1.5 × IQR; points represent individual observations. (c) Seasonal variation in invasive cover within each habitat type, displayed as faceted boxplots across the six habitats. (d) Heatmap showing the predicted mean invasive cover (%) for each habitat‐season combination, with color intensity corresponding to the cover level.

Invasive plant cover was influenced by the interaction between habitat type and season (habitat × season: $\chi_{15}^{2}$ = 30.32, *p* =0.011), while the main effects of habitat ($\chi_{5}^{2}$ = 9.70, *p* = 0.084) and season ($\chi_{3}^{2}$ = 0.84, *p* = 0.840) were not statistically significant. This indicates that seasonal variation in invasive cover differed among habitat types. Specifically, the seasonal patterns varied markedly across habitats: Forest edge showed a significant decline in invasive cover from spring to summer (Estimate = −1.26, *p* = 0.016), Park exhibited a significant decline from spring to summer (Estimate = −1.55, *p* = 0.002), and wetland also showed a significant decline from spring to summer (Estimate = −1.22, *p* = 0.013). In contrast, the roadside and wasteland showed no significant seasonal changes (*p* > 0.05 for all seasonal comparisons), indicating that these highly disturbed habitats maintain consistently high invasion levels throughout the year.

Post hoc comparisons revealed that wasteland, roadside, and forest edge exhibited significantly higher invasive cover than forest habitats (Tukey‐adjusted *p* < 0.05 for all three comparisons; Figure 4b), while park and wetland showed intermediate levels that did not differ significantly from forest (*p* > 0.05 for both). Based on the model estimates and observed distributions (Figure 4b,d), invasive cover was most extensive in wasteland and roadside, where predicted values generally ranged between 50% and 65%. Intermediate cover levels (roughly 25%–50%) were observed in forest edge, park, and wetland, while forest habitats consistently exhibited the lowest invasion levels, typically below 20%.

As shown in the heatmap (Figure 4d), roadside and wasteland maintained consistently high invasion levels across all seasons (ranging from 50% to 68%), while forest remained consistently low (ranging from 9% to 52%, with the lowest values in winter). The forest edge, park, and wetland showed more pronounced seasonal fluctuations, with generally higher cover in autumn and winter than in spring and summer. These patterns indicate that habitat identity exerted a stronger overall influence than seasonal variation on invasion patterns, but seasonal effects were non‐negligible in certain habitat types.

The continuous disturbance index (PC1), when included as a covariate, was also significantly associated with invasive plant cover, corroborating the habitat‐level patterns and indicating that invasion levels covary with both categorical habitat attributes and the intensity of anthropogenic pressure (see Table S1).

Native community patterns inversely mirrored the invasion gradient. Native richness was highest in forest (34.1 ± 9.2) and lowest in forest edge (11.2 ± 4.7) and roadside (11.5 ± 3.6). Similarly, native cover peaked in forest (78.6% ± 8.9%) and wetland (66.3% ± 9.3%), but dropped to approximately 37% in forest edge and park (see Figure S2, Table S6). A negative association was observed between native richness and invasive cover (see Figure S3).

## 4 Discussion

### 4.1 Habitat Specificity and Anthropogenic Disturbance Shape Community Composition

Our results show that habitat‐specific environmental conditions are associated with invasive plant community composition in Hengqin. The PERMANOVA results ($R^{2}$ = 0.589 for habitat vs. $R^{2}$ = 0.012 for season) and the dbRDA marginal tests (habitat: *F* = 3.31, *p* = 0.001; disturbance: *F* = 4.00, *p* = 0.001; season: *F* = 0.64, *p* = 0.955) indicate that habitat type and disturbance intensity are the primary factors associated with community composition. In contrast, the main effect of seasonal variation on overall community composition was not statistically significant, whereas seasonal variation exerts negligible influence.

The NMDS ordination revealed clear habitat‐specific clustering. Roadside and wasteland not only supported the highest invasive cover but also harbored distinct species assemblages (Figure 2a). This pattern aligns with the premise that severe anthropogenic disturbance compromises native biotic resistance and creates physical openings that are rapidly colonized by fast‐growing, disturbance‐adapted invasive species (Hobbs and Huenneke 1992; Catford et al. 2009). Conversely, the consistently low invasion levels in forest habitats suggest that intact native vegetation and specific environmental constraints (e.g., dense canopy shading) likely act as a robust filter, limiting invasion to a subset of shade‐tolerant or flood‐tolerant species regardless of propagule pressure (Levine et al. 2004; Martin et al. 2009).

Notably, the NMDS ordination (Figure 2a) also identified highly isolated clustering of Park habitats, distinct from both natural ecosystems and other disturbed areas. Unlike Wastelands, where succession is spontaneous, Parks are subject to directed assembly through human management (e.g., mowing, weeding) (Kueffer et al. 2010; Williams et al. 2015). This creates a distinct selective environment that appears to favor trampling‐tolerant and mowing‐resilient species, differentiating the park community from other high‐disturbance habitats (Gaertner et al. 2017; Pyšek et al. 2020). Furthermore, the dbRDA biplot (Figure 2c) highlighted the unique divergence of Roadside habitats along the first axis, likely reflecting the extreme stress of traffic emissions and soil compaction. The parallel significance of both habitat and PC1 in the marginal tests reflects the inherent covariation between habitat type and disturbance intensity in our observational design. Together, these findings underscore that in urban ecosystems, disturbance is not a monolithic gradient; rather, the specific type of disturbance (e.g., mechanical maintenance in parks vs. traffic‐related degradation along roadsides) differentially filters species pools (Hulme 2003; Kowarik 2011).

### 4.2 Habitat‐Specific Habitat Occupancy and Species Co‐Occurrence

The dominance of invasive species in our study area is closely linked to their broad spatial distribution patterns. Our finding that invasive species possess significantly higher habitat occupancy breadths than natives (Shannon index: 3.00 vs. 2.45; Figure 3b) supports the generalist strategist hypothesis (Richards et al. 2006; Vázquez 2006). This broader habitat occupancy allows invasive species to occur across a wider range of habitat‐season combinations across the urban landscape, potentially giving them a clear advantage over the more narrowly distributed native species in colonizing disturbed areas (Slatyer et al. 2013).

Our analysis of spatial co‐occurrence provides further insight into how these communities are structured. Invasive species showed a much higher co‐occurrence with each other (median = 0.203) than native species did among themselves (median = 0.032). Although the absolute co‐occurrence values are relatively low (below 0.25), the significant difference between groups is important. In highly disturbed habitats, this pattern suggests habitat‐mediated co‐occurrence associated with environmental filtering. Severe disturbance may favor a specific group of species (e.g., *B. alba* , *M. micrantha* ) that share similar traits for rapid growth and survival (Kraft et al. 2015; Mayfield and Levine 2010). As a result, these invasive species tend to co‐occur and dominate the same disturbed patches, suggesting that environmental filtering may outweigh competitive exclusion in these habitats (Simberloff and Von Holle 1999; Gallien and Carboni 2017).

Finally, the intermediate co‐occurrence between invasive and native species (median = 0.144) is a key finding. This co‐occurrence is significantly higher than the baseline among native species, indicating that invasive species are occupying the spatial domains used by indigenous plants. Combined with their broader occupancy and higher abundance, even a moderate level of co‐occurrence suggests that invasive species may be exerting spatial pressures on native communities (Vilà and Weiner 2004). This suggests that invasive species are not just filling empty spaces but are overlapping in occupancy with native species, which could lead to a long‐term decline in native biodiversity in urban habitats.

### 4.3 Spatial Factors Appear to Predominate Over Seasonality

A notable observation in this study is the relative stability of community composition (Figure 2b) across seasons, as well as the consistent ranking of habitat types in terms of invasive cover (Figure 4c). However, while overall community composition remained stable across seasons, our ZOIB model revealed a significant interaction between habitat type and season ($\chi_{15}^{2}$ = 30.32, *p* = 0.011). The indicates that seasonal variation d significantly influences the magnitude of invasion (e.g., invasive cover), but in a habitat‐specific manner. This nuanced pattern contrasts with many temperate invasion models, where distinct phenological windows, such as spring pulses or autumn senescence, often influence invasion dynamics across entire landscapes (Wolkovich and Cleland 2011; Godoy et al. 2009).

These observed habitat‐dependent seasonal patterns may be attributed to the interaction between the subtropical climate and the life‐history traits of the local invasive flora. The mild winters and consistent precipitation in Hengqin likely reduce the physiological stressors that typically induce seasonal turnover in higher latitudes. Such climatic conditions may allow invasive species to maintain vegetative growth and reproductive activity throughout much of the year (Sakai et al. 2001). However, the significant seasonal declines observed in forest edge, park, and wetland from spring to summer (*p* < 0.05 for all three) suggest that seasonal environmental changes (such as increased shading from canopy development or altered soil moisture) may temporarily reduce invasion in these habitats. Furthermore, many of the dominant invasive species in our study system, such as *B. alba* and *M. micrantha* , exhibit high ecological plasticity and the ability for year‐round persistence in disturbed environments (Pyšek and Richardson 2010). This likely explains why roadside and wasteland maintained consistently high invasion levels across all seasons (50%–68%), as these species are preadapted to persistent disturbance regimes.

Consequently, our results suggest that in this subtropical urban context, the spatial opportunity created by disturbance may be a more consistently influential factor of invasion than temporal windows (Shea and Chesson 2002). However, the significant habitat × season interaction indicates that seasonal effects are non‐negligible in certain habitat types. This implies that invasion risk remains relatively constant year‐round in highly disturbed patches (roadside, wasteland), but may fluctuate seasonally in habitats with more pronounced environmental variation (forest edge, park, wetland). Therefore, management efforts might be most effective if they prioritize the restoration of specific high‐risk habitats while also considering seasonal timing in habitats where fluctuations are observed (Rejmánek and Richardson 1996).

The inverse relationship between native community attributes and invasion intensity across habitats provides additional context for understanding these patterns. Native species richness was highest in forest (34.1 ± 9.18) and lowest in forest edge (11.2 ± 4.65) and roadside (11.5 ± 3.55), while native cover was highest in forest (78.6% ± 8.85%) and wetland (66.3% ± 9.33%), and lowest in forest edge (37.7% ± 26.2%) and park (37.3% ± 15.6%). Forest habitats, which harbored the highest native richness and cover, were consistently the least invaded, whereas roadside and wasteland, with depauperate native communities, supported the highest invasive cover. This pattern aligns with the biotic resistance hypothesis (Levine et al. 2004), suggesting that diverse native communities may exert a filtering effect against invasion. However, given the observational nature of our study, this interpretation remains correlational rather than causal. The concurrent decline of native diversity and increase in invasion along the disturbance gradient further highlights the compounded ecological costs of anthropogenic habitat modification in urban landscapes. Several limitations should be acknowledged. Spatial replication was limited (*n* = 3 plots per habitat), and seasonal measurements are temporal rather than independent spatial replicates. Our observational design cannot disentangle habitat effects from disturbance intensity, and direct environmental variables were not measured, so mechanistic interpretations remain speculative. The disturbance index was semiquantitative, though inter‐observer agreement was acceptable (κ = 0.556 for trampling, κ = 0.874 for human activity). Finally, our findings are restricted to Hengqin Island (ca. 106 km^2^) and require cautious extrapolation. Despite these caveats, this study provides a comprehensive empirical assessment of invasion patterns across major urban habitat types in a subtropical island landscape.

## 5 Conclusion

Our study shows that invasive plant communities in the subtropical urban landscape of Hengqin are closely associated with habitat‐specific environmental conditions and anthropogenic disturbance, while seasonal variation plays a secondary but habitat‐dependent role in shaping invasion patterns. Invasive species consistently exhibited broader habitat occupancy breadths and higher spatial co‐occurrence compared to native species, indicating greater ecological plasticity and a tendency for spatial convergence among invasive species in disturbed environments. The consistently high invasive cover in roadside and wasteland habitats across all seasons, contrasted with seasonal fluctuations in forest edge, park, and wetland habitats, suggests that spatial opportunities, specifically the availability of disturbed sites, may play a more consistently influential role in invasion success than seasonal timing in this subtropical system, although seasonal effects are non‐negligible in certain habitat types. These findings imply that monitoring and management efforts could usefully prioritize high‐risk habitats such as roadside and wasteland habitats, while also considering seasonal timing in habitats where fluctuations are observed. Future research with expanded spatial sampling and direct measurements of environmental variables would help to further clarify the mechanisms underlying the observed patterns within these urban plant communities.

## Author Contributions

**Sha Wang:** data curation (equal), investigation (equal), methodology (equal), software (equal), validation (equal), visualization (equal), writing – original draft (equal). **Ming Li:** investigation (supporting), resources (supporting). **Yanan Guo:** investigation (supporting), resources (supporting). **Jun Li:** investigation (supporting), resources (supporting). **Ruijiang Wang:** data curation (lead), funding acquisition (lead), project administration (lead), supervision (lead), writing – review and editing (lead).

## Funding

This work was supported by the Science & Technology Fundamental Resources Investigation Program (Grant No. 2022FY100501), the Urban Planning and Construction Bureau of the Guangdong‐Macao In‐Depth Cooperation Zone in Hengqin's Background Survey Project of Plant and Animal Resources (Grant No. GDZX2022‐F032W), and the Biodiversity Assessment and Conservation Planning Project (Grant No. HXD230307013).

## Conflicts of Interest

The authors declare no conflicts of interest.

## Supporting information


**Figure S1:** Disturbance indicators across habitat types.
**Figure S2:** Native species cover by habitat.
**Figure S3:** Native richness versus invasive cover.
**Table S1:** PCA loadings and disturbance index (PC1) for the three disturbance indicators.
**Table S2:** Cover data for all vascular plant species recorded across the 72 sampling units.
**Table S3:** Results of the zero–one inflated Beta (ZOIB) mixed‐effects model.
**Table S4:** Species composition, origin, life form and alien extent of invasive plants recorded in all quadrat.
**Table S5:** Summary of the 15 dominant species (seven invasive and eight native) selected for occupancy analysis.
**Table S6:** Native species richness and cover (mean ± SD) across the six habitat types.

## Data Availability

All data generated or analyzed during this study are included in this published article and its Supporting Information. These files have been uploaded to the journal's submission system and are accessible to the editors and reviewers for peer review purposes.
