# Supplementary material for: Habitat Type and Disturbance Intensity Are Associated With Invasive Plant Community Structure Across an Urbanizing Subtropical Island
Source: Ecol Evol. 2026 Oct 20;16(10):e74522. doi: 10.1002/ece3.74522 (PMC13681247; doi:10.1002/ece3.74522)
Supplement: Supplementary file 1 [file ECE3-16-e74522-s001.docx]

**Supplementary Materials**

**Supplementary Materials**

FIGURE S1 Disturbance indicators across habitat types

FIGURE S2 Native species cover by habitat

FIGURE S3 Native richness vs invasive cover

TABLE S1 PCA loadings and disturbance index (PC1) for the three disturbance indicators

| PC1 | PC2 | PC3 | variable |
| --- | --- | --- | --- |
| 0.449823 | -0.88929 | -0.08262 | Road_Distance |
| -0.62268 | -0.37859 | 0.684789 | Trampling |
| -0.64025 | -0.25659 | -0.72404 | Human_Activity |

TABLE S2 Cover data for all vascular plant species recorded across the 72 sampling units

| species | occurrence | mean_cover | total_cover | status | rel_cover_invasive | rel_cover_native |
| --- | --- | --- | --- | --- | --- | --- |
| *Bidens alba* | 0.986111 | 13.92742 | 1002.774 | Invasive | 33.997 | NA |
| *Sphagneticola trilobata* | 0.430556 | 6.174405 | 444.5572 | Invasive | 15.0718 | NA |
| *Leucaena leucocephala* | 0.666667 | 4.608353 | 331.8014 | Invasive | 11.24905 | NA |
| *Mikania micrantha* | 0.597222 | 3.960906 | 285.1853 | Invasive | 9.66862 | NA |
| *Cynodon dactylon* | 0.444444 | 3.091493 | 222.5875 | Native | NA | 6.170778 |
| *Phragmites australis* | 0.347222 | 2.487865 | 179.1263 | Native | NA | 4.965906 |
| *Litchi chinensis* | 0.055556 | 2.30979 | 166.3049 | Native | NA | 4.61046 |
| *Melastoma candidum* | 0.055556 | 2.191242 | 157.7694 | Native | NA | 4.373831 |
| *Saccharum arundinaceum* | 0.319444 | 2.175945 | 156.6681 | Native | NA | 4.343299 |
| *Ficus concinna* | 0.222222 | 2.092161 | 150.6356 | Native | NA | 4.176061 |
| *Acacia confusa* | 0.208333 | 2.000381 | 144.0275 | Native | NA | 3.992864 |
| *Ruellia simplex* | 0.25 | 1.955068 | 140.7649 | Native | NA | 3.902417 |
| *Apluda mutica* | 0.416667 | 1.895465 | 136.4735 | Native | NA | 3.783445 |
| *Panicum repens* | 0.291667 | 1.808707 | 130.2269 | Invasive | 4.415076 | NA |
| *Coccinia grandis* | 0.291667 | 1.663369 | 119.7626 | Native | NA | 3.320171 |
| *Brachiaria mutica* | 0.055556 | 1.651511 | 118.9088 | Invasive | 4.031359 | NA |
| *Lantana camara* | 0.527778 | 1.586303 | 114.2138 | Invasive | 3.872185 | NA |
| *Casuarina equisetifolia* | 0.055556 | 1.546644 | 111.3584 | Native | NA | 3.087182 |
| *Sesbania cannabina* | 0.555556 | 1.511221 | 108.8079 | Invasive | 3.688909 | NA |
| *Sonneratia apetala* | 0.097222 | 1.439079 | 103.6137 | Invasive | 3.512809 | NA |
| *Wollastonia biflora* | 0.263889 | 1.395217 | 100.4557 | Native | NA | 2.784926 |
| *Pueraria montana var. lobata* | 0.319444 | 1.339385 | 96.43571 | Native | NA | 2.673481 |
| *Neyraudia reynaudiana* | 0.361111 | 1.270649 | 91.48674 | Native | NA | 2.536281 |
| *Eriochloa procera* | 0.388889 | 1.222534 | 88.02243 | Native | NA | 2.44024 |
| *Ipomoea pes-caprae* | 0.166667 | 1.213308 | 87.35815 | Native | NA | 2.421824 |
| *Ipomoea aquatica* | 0.166667 | 1.175547 | 84.63936 | Native | NA | 2.346451 |
| *Typha angustifolia* | 0.111111 | 1.007615 | 72.54829 | Native | NA | 2.011251 |
| *Litsea glutinosa* | 0.166667 | 0.940867 | 67.74242 | Native | NA | 1.878019 |
| *Myriophyllum spicatum* | 0.055556 | 0.670608 | 48.2838 | Native | NA | 1.338569 |
| *Talipariti tiliaceum* | 0.166667 | 0.659872 | 47.51082 | Native | NA | 1.317139 |
| *Symphyotrichum subulatum* | 0.444444 | 0.644925 | 46.43459 | Invasive | 1.574269 | NA |
| *Dicranopteris pedata* | 0.111111 | 0.637364 | 45.89024 | Native | NA | 1.272212 |
| *Melaleuca viminalis* | 0.055556 | 0.563631 | 40.58144 | Native | NA | 1.125037 |
| *Paederia foetida* | 0.402778 | 0.554595 | 39.93087 | Native | NA | 1.107001 |
| *Fimbristylis cymosa* | 0.305556 | 0.55199 | 39.7433 | Native | NA | 1.101801 |
| *Averrhoa carambola* | 0.055556 | 0.533029 | 38.37806 | Native | NA | 1.063952 |
| *Mimosa diplotricha var. inermis* | 0.194444 | 0.513462 | 36.96924 | Invasive | 1.253366 | NA |
| *Fimbristylis sieboldii* | 0.25 | 0.473624 | 34.10093 | Native | NA | 0.945378 |
| *Ipomoea cairica* | 0.152778 | 0.454794 | 32.74517 | Invasive | 1.110158 | NA |
| *Celtis sinensis* | 0.125 | 0.448492 | 32.29141 | Native | NA | 0.895213 |
| *Camphora officinarum* | 0.041667 | 0.423051 | 30.45966 | Native | NA | 0.844431 |
| *Merremia hederacea* | 0.291667 | 0.420472 | 30.274 | Native | NA | 0.839284 |
| *Mimosa pudica* | 0.5 | 0.419442 | 30.19983 | Invasive | 1.023863 | NA |
| *Miscanthus sinensis* | 0.069444 | 0.40857 | 29.41705 | Native | NA | 0.815527 |
| *Alocasia odora* | 0.111111 | 0.380726 | 27.41229 | Native | NA | 0.759949 |
| *Delonix regia* | 0.055556 | 0.355352 | 25.58537 | Native | NA | 0.709302 |
| *Ficus variolosa* | 0.055556 | 0.346755 | 24.96638 | Native | NA | 0.692141 |
| *Toxicodendron succedaneum* | 0.111111 | 0.328236 | 23.63298 | Native | NA | 0.655176 |
| *Bougainvillea spectabilis* | 0.111111 | 0.302392 | 21.7722 | Native | NA | 0.603589 |
| *Triadica cochinchinensis* | 0.125 | 0.294177 | 21.18072 | Native | NA | 0.587192 |
| *Pennisetum polystachion* | 0.111111 | 0.290111 | 20.88796 | Invasive | 0.708163 | NA |
| *Eucalyptus robusta* | 0.069444 | 0.290092 | 20.88661 | Native | NA | 0.579038 |
| *Chloris virgata* | 0.263889 | 0.273972 | 19.72597 | Native | NA | 0.546862 |
| *Melinis repens* | 0.263889 | 0.238438 | 17.1675 | Invasive | 0.582029 | NA |
| *Canna indica* | 0.055556 | 0.235839 | 16.98043 | Native | NA | 0.470747 |
| *Acacia auriculiformis* | 0.125 | 0.235188 | 16.93353 | Native | NA | 0.469447 |
| *Laguncularia racemosa* | 0.055556 | 0.225452 | 16.23258 | Native | NA | 0.450015 |
| *Acrostichum aureum* | 0.166667 | 0.217362 | 15.65007 | Native | NA | 0.433866 |
| *Ipomoea triloba* | 0.305556 | 0.214904 | 15.47309 | Invasive | 0.524583 | NA |
| *Bothriochloa bladhii* | 0.138889 | 0.210414 | 15.1498 | Native | NA | 0.419997 |
| *Pluchea indica* | 0.111111 | 0.208243 | 14.99352 | Native | NA | 0.415664 |
| *Alternanthera philoxeroides* | 0.291667 | 0.200103 | 14.40743 | Invasive | 0.488454 | NA |
| *Bauhinia × blakeana* | 0.041667 | 0.200087 | 14.40624 | Native | NA | 0.399383 |
| *Lagerstroemia speciosa* | 0.041667 | 0.196537 | 14.15064 | Native | NA | 0.392297 |
| *Miscanthus floridulus* | 0.138889 | 0.186877 | 13.45514 | Native | NA | 0.373016 |
| *Sinosideroxylon wightianum* | 0.111111 | 0.184747 | 13.30181 | Native | NA | 0.368765 |
| *Ficus subpisocarpa* | 0.083333 | 0.181251 | 13.05005 | Native | NA | 0.361786 |
| *Ilex rotunda* | 0.055556 | 0.173378 | 12.48319 | Native | NA | 0.346071 |
| *Pinus massoniana* | 0.055556 | 0.173378 | 12.48319 | Native | NA | 0.346071 |
| *Atalantia buxifolia* | 0.055556 | 0.171849 | 12.37315 | Native | NA | 0.34302 |
| *Canavalia rosea* | 0.083333 | 0.13895 | 10.0044 | Native | NA | 0.277351 |
| *Crotalaria pallida* | 0.125 | 0.138449 | 9.968328 | Invasive | 0.337956 | NA |
| *Eleusine indica* | 0.472222 | 0.137356 | 9.889655 | Native | NA | 0.27417 |
| *Zanthoxylum avicennae* | 0.111111 | 0.13559 | 9.762497 | Native | NA | 0.270645 |
| *Cyperus surinamensis* | 0.25 | 0.133322 | 9.599157 | Invasive | 0.32544 | NA |
| *Grona heterocarpos* | 0.208333 | 0.126342 | 9.096597 | Native | NA | 0.252184 |
| *Bridelia tomentosa* | 0.111111 | 0.124199 | 8.94232 | Native | NA | 0.247907 |
| *Cyperus rotundus* | 0.208333 | 0.122442 | 8.815817 | Native | NA | 0.2444 |
| *Dactyloctenium aegyptium* | 0.194444 | 0.121414 | 8.741832 | Native | NA | 0.242349 |
| *Tamarix chinensis* | 0.097222 | 0.116851 | 8.413291 | Native | NA | 0.233241 |
| *Rhododendron × pulchrum* | 0.055556 | 0.115585 | 8.322126 | Native | NA | 0.230714 |
| *Rhus chinensis* | 0.055556 | 0.115585 | 8.322126 | Native | NA | 0.230714 |
| *Heptapleurum heptaphyllum* | 0.069444 | 0.11234 | 8.088474 | Native | NA | 0.224236 |
| *Livistona chinensis* | 0.055556 | 0.111514 | 8.028987 | Native | NA | 0.222587 |
| *Mallotus paniculatus* | 0.055556 | 0.106606 | 7.675611 | Native | NA | 0.21279 |
| *Ricinus communis* | 0.041667 | 0.105372 | 7.5868 | Invasive | 0.257215 | NA |
| *Archidendron clypearia* | 0.055556 | 0.105058 | 7.564181 | Native | NA | 0.209701 |
| *Rhodomyrtus tomentosa* | 0.055556 | 0.105058 | 7.564181 | Native | NA | 0.209701 |
| *Schima superba* | 0.055556 | 0.105058 | 7.564181 | Native | NA | 0.209701 |
| *Desmodium tortuosum* | 0.125 | 0.101658 | 7.319345 | Invasive | 0.248147 | NA |
| *Solanum americanum* | 0.347222 | 0.098692 | 7.105837 | Native | NA | 0.196995 |
| *Rubus reflexus* | 0.041667 | 0.098407 | 7.085287 | Native | NA | 0.196425 |
| *Cyperus odoratus* | 0.236111 | 0.09794 | 7.051715 | Native | NA | 0.195494 |
| *Senna occidentalis* | 0.041667 | 0.096766 | 6.96714 | Invasive | 0.236207 | NA |
| *Cyclosorus interruptus* | 0.083333 | 0.093066 | 6.700721 | Native | NA | 0.185764 |
| *Cyperus polystachyos* | 0.069444 | 0.092851 | 6.685274 | Native | NA | 0.185335 |
| *Echinochloa colona* | 0.069444 | 0.088558 | 6.376175 | Native | NA | 0.176766 |
| *Passiflora foetida* | 0.138889 | 0.087045 | 6.267253 | Invasive | 0.212478 | NA |
| *Solanum torvum* | 0.111111 | 0.086574 | 6.233324 | Invasive | 0.211328 | NA |
| *Trema tomentosa* | 0.083333 | 0.08558 | 6.161744 | Native | NA | 0.170822 |
| *Euphorbia hirta* | 0.236111 | 0.085064 | 6.124593 | Invasive | 0.207642 | NA |
| *Urena lobata* | 0.125 | 0.082834 | 5.964054 | Native | NA | 0.165341 |
| *Ischaemum aristatum* | 0.055556 | 0.080415 | 5.78988 | Native | NA | 0.160512 |
| *Eclipta prostrata* | 0.25 | 0.076458 | 5.50498 | Native | NA | 0.152614 |
| *Rhaphiolepis indica* | 0.111111 | 0.073175 | 5.268625 | Native | NA | 0.146062 |
| *Melastoma sanguineum* | 0.125 | 0.07276 | 5.238714 | Native | NA | 0.145233 |
| *Embelia laeta* | 0.111111 | 0.072314 | 5.20663 | Native | NA | 0.144343 |
| *Mussaenda pubescens* | 0.055556 | 0.072102 | 5.191336 | Native | NA | 0.143919 |
| *Cassytha filiformis* | 0.083333 | 0.070436 | 5.071421 | Native | NA | 0.140595 |
| *Sageretia thea* | 0.055556 | 0.06874 | 4.949262 | Native | NA | 0.137208 |
| *Leptochloa chinensis* | 0.125 | 0.067755 | 4.878347 | Native | NA | 0.135242 |
| *Pluchea sagittalis* | 0.152778 | 0.066045 | 4.755271 | Invasive | 0.161218 | NA |
| *Clerodendrum indicum* | 0.055556 | 0.065518 | 4.717324 | Native | NA | 0.130778 |
| *Sida rhombifolia* | 0.138889 | 0.063923 | 4.602424 | Native | NA | 0.127593 |
| *Rhodoleia championii* | 0.055556 | 0.062678 | 4.512832 | Native | NA | 0.125109 |
| *Digitaria sanguinalis* | 0.152778 | 0.06238 | 4.491382 | Native | NA | 0.124514 |
| *Baeckea frutescens* | 0.055556 | 0.057793 | 4.161063 | Native | NA | 0.115357 |
| *Commelina diffusa* | 0.069444 | 0.057352 | 4.129331 | Native | NA | 0.114477 |
| *Volkameria inermis* | 0.013889 | 0.057245 | 4.121672 | Native | NA | 0.114265 |
| *Hydrocotyle verticillata* | 0.055556 | 0.056164 | 4.043778 | Invasive | 0.137096 | NA |
| *Blechnopsis orientalis* | 0.125 | 0.054977 | 3.958322 | Native | NA | 0.109736 |
| *Pycreus flavidus* | 0.055556 | 0.052972 | 3.814006 | Native | NA | 0.105735 |
| *Elaeocarpus japonicus* | 0.055556 | 0.050285 | 3.620552 | Native | NA | 0.100372 |
| *Hypserpa nitida* | 0.097222 | 0.047579 | 3.425673 | Native | NA | 0.09497 |
| *Cuscuta campestris* | 0.069444 | 0.047486 | 3.419001 | Invasive | 0.115914 | NA |
| *Cyperus iria* | 0.152778 | 0.047106 | 3.391597 | Native | NA | 0.094025 |
| *Paspalum urvillei* | 0.041667 | 0.045803 | 3.297795 | Invasive | 0.111805 | NA |
| *Asparagus cochinchinensis* | 0.055556 | 0.04508 | 3.245767 | Native | NA | 0.089982 |
| *Celosia argentea* | 0.152778 | 0.044649 | 3.214698 | Native | NA | 0.089121 |
| *Paspalum scrobiculatum var. orbiculare* | 0.236111 | 0.040086 | 2.886195 | Native | NA | 0.080014 |
| *Echinochloa crus-galli var. breviseta* | 0.069444 | 0.039389 | 2.835975 | Native | NA | 0.078622 |
| *Eurya nitida* | 0.111111 | 0.036665 | 2.6399 | Native | NA | 0.073186 |
| *Breynia fruticosa* | 0.055556 | 0.035925 | 2.586623 | Native | NA | 0.071709 |
| *Tridax procumbens* | 0.180556 | 0.035295 | 2.541218 | Invasive | 0.086155 | NA |
| *Adiantum flabellulatum* | 0.055556 | 0.035019 | 2.521394 | Native | NA | 0.0699 |
| *Callerya nitida* | 0.055556 | 0.035019 | 2.521394 | Native | NA | 0.0699 |
| *Diplospora dubia* | 0.055556 | 0.035019 | 2.521394 | Native | NA | 0.0699 |
| *Gardenia jasminoides* | 0.055556 | 0.035019 | 2.521394 | Native | NA | 0.0699 |
| *Itea chinensis* | 0.055556 | 0.035019 | 2.521394 | Native | NA | 0.0699 |
| *Smilax japonica* | 0.055556 | 0.035019 | 2.521394 | Native | NA | 0.0699 |
| *Syzygium kwangtungense* | 0.055556 | 0.035019 | 2.521394 | Native | NA | 0.0699 |
| *Glochidion puberum* | 0.041667 | 0.034172 | 2.460414 | Native | NA | 0.06821 |
| *Gnetum montanum* | 0.055556 | 0.034128 | 2.457216 | Native | NA | 0.068121 |
| *Leptochloa panicea* | 0.013889 | 0.033776 | 2.431907 | Native | NA | 0.06742 |
| *Ipomoea purpurea* | 0.097222 | 0.031399 | 2.260756 | Invasive | 0.076646 | NA |
| *Amaranthus viridis* | 0.138889 | 0.028882 | 2.079477 | Invasive | 0.0705 | NA |
| *Arundo donax* | 0.041667 | 0.02858 | 2.057735 | Native | NA | 0.057046 |
| *Melochia corchorifolia* | 0.013889 | 0.028027 | 2.017937 | Native | NA | 0.055943 |
| *Bombax ceiba* | 0.041667 | 0.027481 | 1.978621 | Native | NA | 0.054853 |
| *Lophatherum gracile* | 0.041667 | 0.027018 | 1.945308 | Native | NA | 0.05393 |
| *Psychotria serpens* | 0.097222 | 0.026201 | 1.886484 | Native | NA | 0.052299 |
| *Cyperus cyperoides* | 0.111111 | 0.024731 | 1.780644 | Native | NA | 0.049365 |
| *Centella asiatica* | 0.111111 | 0.024526 | 1.765849 | Native | NA | 0.048955 |
| *Alysicarpus vaginalis* | 0.069444 | 0.02438 | 1.755353 | Native | NA | 0.048664 |
| *Youngia japonica* | 0.083333 | 0.022662 | 1.631656 | Native | NA | 0.045234 |
| *Lygodium japonicum* | 0.083333 | 0.021287 | 1.532667 | Native | NA | 0.04249 |
| *Euphorbia prostrata* | 0.111111 | 0.021063 | 1.516562 | Invasive | 0.051416 | NA |
| *Phyllanthus urinaria* | 0.208333 | 0.020896 | 1.504482 | Native | NA | 0.041709 |
| *Asystasia gangetica subsp. Micrantha* | 0.041667 | 0.020482 | 1.474729 | Invasive | 0.049998 | NA |
| *Euphorbia hypericifolia* | 0.166667 | 0.019846 | 1.428909 | Invasive | 0.048444 | NA |
| *Carica papaya* | 0.013889 | 0.019703 | 1.418641 | Native | NA | 0.039329 |
| *Ischaemum barbatum* | 0.027778 | 0.01913 | 1.377337 | Native | NA | 0.038184 |
| *Dendrotrophe varians* | 0.055556 | 0.018991 | 1.367359 | Native | NA | 0.037907 |
| *Praxelis clematidea* | 0.152778 | 0.018939 | 1.363623 | Invasive | 0.046231 | NA |
| *Emilia sonchifolia* | 0.277778 | 0.018157 | 1.307299 | Native | NA | 0.036242 |
| *Cyrtococcum patens var. latifolium* | 0.041667 | 0.018012 | 1.296839 | Native | NA | 0.035952 |
| *Eragrostis pilosa* | 0.055556 | 0.017859 | 1.285862 | Native | NA | 0.035648 |
| *Oxalis corniculata* | 0.194444 | 0.017711 | 1.275173 | Native | NA | 0.035352 |
| *Glochidion wrightii* | 0.055556 | 0.01751 | 1.260697 | Native | NA | 0.03495 |
| *Morus alba* | 0.055556 | 0.017282 | 1.244309 | Native | NA | 0.034496 |
| *Phoenix loureiroi* | 0.013889 | 0.017065 | 1.228669 | Native | NA | 0.034062 |
| *Gymnema sylvestre* | 0.041667 | 0.016479 | 1.186516 | Native | NA | 0.032894 |
| *Cyperus eragrostis* | 0.027778 | 0.01531 | 1.102338 | Native | NA | 0.03056 |
| *Lindernia ruellioides* | 0.027778 | 0.015229 | 1.096491 | Native | NA | 0.030398 |
| *Jasminum elongatum* | 0.041667 | 0.015059 | 1.084277 | Native | NA | 0.030059 |
| *Bischofia javanica* | 0.041667 | 0.01482 | 1.06705 | Native | NA | 0.029582 |
| *Phragmites karka* | 0.013889 | 0.014778 | 1.063981 | Native | NA | 0.029497 |
| *Macroptilium lathyroides* | 0.069444 | 0.014773 | 1.063656 | Invasive | 0.036061 | NA |
| *Aporosa dioica* | 0.041667 | 0.0135 | 0.972026 | Native | NA | 0.026947 |
| *Triumfetta rhomboidea* | 0.041667 | 0.013251 | 0.954097 | Invasive | 0.032347 | NA |
| *Oldenlandia corymbosa* | 0.097222 | 0.013157 | 0.947335 | Native | NA | 0.026263 |
| *Rhynchospora rubra* | 0.013889 | 0.013134 | 0.945626 | Native | NA | 0.026216 |
| *Pycreus sanguinolentus* | 0.083333 | 0.012599 | 0.907132 | Native | NA | 0.025148 |
| *Macroptilium atropurpureum* | 0.041667 | 0.012457 | 0.896877 | Invasive | 0.030407 | NA |
| *Lindernia crustacea* | 0.097222 | 0.012444 | 0.895932 | Native | NA | 0.024838 |
| *Aster ageratoides* | 0.055556 | 0.011576 | 0.833492 | Native | NA | 0.023107 |
| *Cajanus scarabaeoides* | 0.013889 | 0.011377 | 0.819113 | Native | NA | 0.022708 |
| *Pennisetum purpureum* | 0.013889 | 0.010711 | 0.771208 | Invasive | 0.026146 | NA |
| *Alyxia sinensis* | 0.055556 | 0.010506 | 0.756418 | Native | NA | 0.02097 |
| *Cyperus difformis* | 0.027778 | 0.010082 | 0.725913 | Native | NA | 0.020124 |
| *Sporobolus fertilis* | 0.041667 | 0.009922 | 0.714359 | Native | NA | 0.019804 |
| *Sterculia lanceolata* | 0.013889 | 0.009852 | 0.70932 | Native | NA | 0.019664 |
| *Cerbera manghas* | 0.013889 | 0.009325 | 0.671398 | Native | NA | 0.018613 |
| *Portulaca oleracea* | 0.069444 | 0.008218 | 0.591699 | Native | NA | 0.016404 |
| *Alternanthera sessilis* | 0.041667 | 0.008147 | 0.586595 | Native | NA | 0.016262 |
| *Spermacoce remota* | 0.055556 | 0.007577 | 0.545542 | Invasive | 0.018495 | NA |
| *Kyllinga nemoralis* | 0.041667 | 0.007461 | 0.537158 | Native | NA | 0.014892 |
| *Echinochloa crus-galli* | 0.013889 | 0.007453 | 0.536625 | Native | NA | 0.014877 |
| *Eragrostis ferruginea* | 0.069444 | 0.007437 | 0.535483 | Native | NA | 0.014845 |
| *Stachytarpheta jamaicensis* | 0.069444 | 0.007048 | 0.507466 | Invasive | 0.017205 | NA |
| *Citrus japonica* | 0.055556 | 0.007004 | 0.504279 | Native | NA | 0.01398 |
| *Rubus leucanthus* | 0.055556 | 0.007004 | 0.504279 | Native | NA | 0.01398 |
| *Duranta erecta* | 0.041667 | 0.006919 | 0.498141 | Native | NA | 0.01381 |
| *Physalis angulata* | 0.041667 | 0.00678 | 0.488133 | Invasive | 0.016549 | NA |
| *Cyperus haspan* | 0.013889 | 0.006755 | 0.486381 | Native | NA | 0.013484 |
| *Malvastrum coromandelianum* | 0.027778 | 0.006226 | 0.448244 | Invasive | 0.015197 | NA |
| *Pteris vittata* | 0.097222 | 0.005785 | 0.416521 | Native | NA | 0.011547 |
| *Kyllinga polyphylla* | 0.069444 | 0.005664 | 0.40784 | Native | NA | 0.011307 |
| *Ageratum conyzoides* | 0.111111 | 0.005567 | 0.40081 | Invasive | 0.013589 | NA |
| *Plantago major* | 0.041667 | 0.005501 | 0.396071 | Native | NA | 0.01098 |
| *Dianella ensifolia* | 0.027778 | 0.005387 | 0.387832 | Native | NA | 0.010752 |
| *Scoparia dulcis* | 0.069444 | 0.004693 | 0.33787 | Invasive | 0.011455 | NA |
| *Brachiaria subquadripara* | 0.013889 | 0.004599 | 0.331097 | Native | NA | 0.009179 |
| *Celastrus hindsii* | 0.041667 | 0.004521 | 0.325486 | Native | NA | 0.009023 |
| *Cardamine occulta* | 0.013889 | 0.004516 | 0.325172 | Native | NA | 0.009015 |
| *Hedyotis caudatifolia* | 0.055556 | 0.004387 | 0.315838 | Native | NA | 0.008756 |
| *Oxybasis glauca* | 0.027778 | 0.004209 | 0.303069 | Native | NA | 0.008402 |
| *Centipeda minima* | 0.041667 | 0.004124 | 0.296895 | Native | NA | 0.008231 |
| *Callerya dielsiana* | 0.027778 | 0.003904 | 0.281123 | Native | NA | 0.007794 |
| *Toddalia asiatica* | 0.041667 | 0.003834 | 0.276037 | Native | NA | 0.007653 |
| *Capillipedium parviflorum* | 0.013889 | 0.003628 | 0.261233 | Native | NA | 0.007242 |
| *Rhynchosia volubilis* | 0.041667 | 0.003387 | 0.243837 | Native | NA | 0.00676 |
| *Cyperus malaccensis* | 0.013889 | 0.003337 | 0.24024 | Native | NA | 0.00666 |
| *Zehneria japonica* | 0.013889 | 0.003337 | 0.24024 | Native | NA | 0.00666 |
| *Corchorus aestuans* | 0.041667 | 0.003332 | 0.239931 | Native | NA | 0.006652 |
| *Panicum maximum* | 0.013889 | 0.003066 | 0.220731 | Invasive | 0.007483 | NA |
| *Scutellaria indica* | 0.041667 | 0.002791 | 0.200958 | Native | NA | 0.005571 |
| *Chrysanthemum indicum* | 0.069444 | 0.002225 | 0.160183 | Native | NA | 0.004441 |
| *Amaranthus spinosus* | 0.013889 | 0.001694 | 0.121939 | Invasive | 0.004134 | NA |
| *Pycreus pumilus* | 0.013889 | 0.001533 | 0.110366 | Native | NA | 0.00306 |
| *Carallia brachiata* | 0.041667 | 0.001325 | 0.09541 | Native | NA | 0.002645 |
| *Wikstroemia indica* | 0.013889 | 0.001238 | 0.089129 | Native | NA | 0.002471 |
| *Ficus hirta* | 0.013889 | 0.001129 | 0.081293 | Native | NA | 0.002254 |
| *Pandanus austrosinensis* | 0.027778 | 0.001124 | 0.080938 | Native | NA | 0.002244 |
| *Lindernia anagallis* | 0.027778 | 0.001109 | 0.079826 | Native | NA | 0.002213 |
| *Setaria viridis* | 0.027778 | 0.001015 | 0.073059 | Native | NA | 0.002025 |
| *Erigeron bonariensis* | 0.041667 | 0.000914 | 0.065779 | Invasive | 0.00223 | NA |
| *Erigeron canadensis* | 0.013889 | 0.000886 | 0.063822 | Invasive | 0.002164 | NA |
| *Artemisia indica* | 0.027778 | 0.000885 | 0.063687 | Native | NA | 0.001766 |
| *Mecardonia procumbens* | 0.027778 | 0.00086 | 0.061948 | Invasive | 0.0021 | NA |
| *Causonis corniculata* | 0.027778 | 0.000813 | 0.058512 | Native | NA | 0.001622 |
| *Phanera championii* | 0.013889 | 0.000733 | 0.052789 | Native | NA | 0.001463 |
| *Parthenocissus semicordata* | 0.041667 | 0.000555 | 0.039976 | Native | NA | 0.001108 |
| *Acalypha australis* | 0.013889 | 0.000452 | 0.032517 | Native | NA | 0.000901 |
| *Ottochloa nodosa var. micrantha* | 0.013889 | 0.000442 | 0.031857 | Native | NA | 0.000883 |
| *Pouzolzia zeylanica* | 0.041667 | 0.000376 | 0.027063 | Native | NA | 0.00075 |
| *Ludwigia prostrata* | 0.013889 | 0.000271 | 0.01951 | Native | NA | 0.000541 |
| *Ilex pubescens* | 0.055556 | 0.000256 | 0.018458 | Native | NA | 0.000512 |
| *Cyanthillium cinereum* | 0.027778 | 0.000212 | 0.015233 | Native | NA | 0.000422 |
| *Stellaria aquatica* | 0.055556 | 0.000198 | 0.014245 | Native | NA | 0.000395 |
| *Ludwigia hyssopifolia* | 0.013889 | 0.000169 | 0.012203 | Native | NA | 0.000338 |
| *Pseudognaphalium affine* | 0.041667 | 0.000133 | 0.009541 | Native | NA | 0.000265 |
| *Hedyotis hedyotidea* | 0.013889 | 0.000132 | 0.009493 | Native | NA | 0.000263 |
| *Parthenocissus chinensis* | 0.013889 | 0.000116 | 0.008344 | Native | NA | 0.000231 |
| *Tephrosia candida* | 0.027778 | 9.17788333333333e-05 | 0.006608 | Invasive | 0.000224 | NA |
| *Emilia praetermissa* | 0.027778 | 9.12958611111111e-05 | 0.006573 | Invasive | 0.000223 | NA |
| *Rorippa cantoniensis* | 0.013889 | 8.14693194444445e-05 | 0.005866 | Native | NA | 0.000163 |
| *Smilax glabra* | 0.013889 | 7.54666805555556e-05 | 0.005434 | Native | NA | 0.000151 |
| *Pilea microphylla* | 0.027778 | 6.97560416666667e-05 | 0.005022 | Invasive | 0.00017 | NA |
| *Operculina turpethum* | 0.013889 | 6.67334027777778e-05 | 0.004805 | Native | NA | 0.000133 |
| *Paspalum conjugatum* | 0.013889 | 6.4048375e-05 | 0.004611 | Invasive | 0.000156 | NA |
| *Bothriospermum zeylanicum* | 0.013889 | 6.06236944444444e-05 | 0.004365 | Native | NA | 0.000121 |
| *Synedrella nodiflora* | 0.013889 | 6.06236944444444e-05 | 0.004365 | Invasive | 0.000148 | NA |
| *Arthraxon hispidus* | 0.013889 | 4.43210555555556e-05 | 0.003191 | Native | NA | 8.8467104564705e-05 |
| *Leonurus japonicus* | 0.013889 | 4.17171388888889e-05 | 0.003004 | Native | NA | 8.32695530817755e-05 |
| *Mazus pumilus* | 0.013889 | 4.13864861111111e-05 | 0.00298 | Native | NA | 8.26095531449597e-05 |
| *Chenopodium album* | 0.013889 | 3.56025e-05 | 0.002563 | Native | NA | 7.1064419625947e-05 |
| *Grona reticulata* | 0.013889 | 3.33666944444444e-05 | 0.002402 | Native | NA | 6.66016368381646e-05 |
| *Camonea pilosa* | 0 | 0 | 0 | Native | NA | 0 |
| *Ceratopteris thalictroides* | 0 | 0 | 0 | Native | NA | 0 |
| *Cyperus compressus* | 0 | 0 | 0 | Native | NA | 0 |
| *Euphorbia thymifolia* | 0 | 0 | 0 | Native | NA | 0 |
| *Oxalis corymbosa* | 0 | 0 | 0 | Invasive | 0 | NA |
| *Scleromitrion diffusum* | 0 | 0 | 0 | Native | NA | 0 |

TABLE S3 Results of the zero-one inflated Beta (ZOIB) mixed-effects model

| parameters | Estimate | Std. Error | z value | P value |
| --- | --- | --- | --- | --- |
| (Intercept) | -2.5227 | 0.7117 | -3.545 | 0.0004 |
| Habitat: Forest edge | 2.7618 | 0.9851 | 2.804 | 0.0051 |
| Habitat: Park | 1.5695 | 0.9847 | 1.594 | 0.1110 |
| Habitat: Roadside | 2.5096 | 0.9757 | 2.572 | 0.0101 |
| Habitat: Wasteland | 2.7621 | 0.9736 | 2.837 | 0.0046 |
| Habitat: Wetland | 1.7914 | 0.9734 | 1.840 | 0.0657 |
| Season: Spring | 0.7339 | 0.3960 | 1.853 | 0.0639 |
| Season: Summer | 0.8019 | 0.3917 | 2.047 | 0.0406 |
| Season: Winter | 0.0470 | 0.4266 | 0.110 | 0.9123 |
| Forest edge × Spring | -1.2572 | 0.5226 | -2.406 | 0.0161 |
| Park × Spring | -1.0109 | 0.5063 | -1.997 | 0.0459 |
| Roadside × Spring | -0.4827 | 0.4916 | -0.982 | 0.3261 |
| Wasteland × Spring | -0.5920 | 0.4838 | -1.224 | 0.2211 |
| Wetland × Spring | -0.7393 | 0.4887 | -1.513 | 0.1303 |
| Forest edge × Summer | -1.2028 | 0.5137 | -2.341 | 0.0192 |
| Park × Summer | -1.5472 | 0.5105 | -3.031 | 0.0024 |
| Roadside × Summer | 0.0969 | 0.4938 | 0.196 | 0.8444 |
| Wasteland × Summer | -0.2958 | 0.4840 | -0.611 | 0.5410 |
| Wetland × Summer | -1.2192 | 0.4923 | -2.477 | 0.0133 |
| Forest edge × Winter | -0.0365 | 0.5391 | -0.068 | 0.9460 |
| Park × Winter | 0.0164 | 0.5308 | 0.031 | 0.9754 |
| Roadside × Winter | 0.7216 | 0.5202 | 1.387 | 0.1654 |

TABLE S4 Species composition, origin, life form and alien extent of invasive plants recorded in all quadrat

| Family | Genus | Latin name | Original region | Life style | Invasive extent | Frequency |
| --- | --- | --- | --- | --- | --- | --- |
| Acanthaceae | *Asystasia* | *Asystasia gangetica subsp. Micrantha* | Africa | Herb | To be observed | 3 |
| Amaranthaceae | *Alternanthera* | *Alternanthera philoxeroides* | South America | Herb | 1 | 3 |
| Amaranthaceae | *Amaranthus* | *Amaranthus spinosus* | South America | Herb | 1 | 2 |
| Amaranthaceae | *Amaranthus* | *Amaranthus viridis* | South America | Herb | 2 | 11 |
| Araliaceae | *Hydrocotyle* | *Hydrocotyle verticillata* | North America | Herb | 2 | 1 |
| Asteraceae | *Erigeron* | *Erigeron canadensis* | North America | Herb | 1 | 1 |
| Asteraceae | *Bidens* | *Bidens alba* | North America | Herb | 1 | 117 |
| Asteraceae | *Ageratum* | *Ageratum conyzoides* | Tropical Americas | Herb | 1 | 4 |
| Asteraceae | *Mikania* | *Mikania micrantha* | Central & South America | Vine | 1 | 59 |
| Asteraceae | *Praxelis* | *Praxelis clematidea* | South America | Herb | 1 | 6 |
| Asteraceae | *Symphyotrichum* | *Symphyotrichum subulatum* | North America | Herb | 1 | 41 |
| Asteraceae | *Erigeron* | *Erigeron bonariensis* | South America | Herb | 2 | 2 |
| Asteraceae | *Synedrella* | *Synedrella nodiflora* | Tropical Americas | Herb | 2 | 1 |
| Asteraceae | *Sphagneticola* | *Sphagneticola trilobata* | Tropical Americas | Herb | 2 | 37 |
| Asteraceae | *Tridax* | *Tridax procumbens* | Americas | Herb | 2 | 14 |
| Asteraceae | *Pluchea* | *Pluchea sagittalis* | South America and the West Indies | Herb | 3 | 15 |
| Asteraceae | *Emilia* | *Emilia praetermissa* | West Africa | Herb | To be observed | 2 |
| Convolvulaceae | *Ipomoea* | *Ipomoea triloba* | North America | Herb | 1 | 20 |
| Convolvulaceae | *Ipomoea* | *Ipomoea cairica* | Tropical Asia and Africa | Herb | 1 | 11 |
| Convolvulaceae | *Ipomoea* | *Ipomoea purpurea* | Americas | Herb | 1 | 7 |
| Convolvulaceae | *Cuscuta* | *Cuscuta campestris* | North America、South America | Vine | 4 | 7 |
| Euphorbiaceae | *Ricinus* | *Ricinus communis* | Tropical East Africa | Herb or Shrub | 2 | 3 |
| Euphorbiaceae | *Euphorbia* | *Euphorbia hirta* | India | Herb | 2 | 20 |
| Euphorbiaceae | *Euphorbia* | *Euphorbia hypericifolia* | North America | Herb | 3 | 12 |
| Euphorbiaceae | *Euphorbia* | *Euphorbia prostrata* | Americas | Herb | 4 | 6 |
| Fabaceae | *Leucaena* | *Leucaena leucocephala* | Americas | Shrub or Tree | 1 | 70 |
| Fabaceae | *Mimosa* | *Mimosa diplotricha var. inermis* | Java | Herb | 2 | 14 |
| Fabaceae | *Mimosa* | *Mimosa pudica* | Tropical Americas | Herb | 2 | 39 |
| Fabaceae | *Desmodium* | *Desmodium tortuosum* | South America and the West Indies | Herb | 3 | 9 |
| Fabaceae | *Macroptilium* | *Macroptilium atropurpureum* | Tropical Americas | Herb | To be observed | 3 |
| Fabaceae | *Macroptilium* | *Macroptilium lathyroides* | Tropical Americas | Herb | To be observed | 3 |
| Fabaceae | *Tephrosia* | *Tephrosia candida* | Eastern Indian Islands and the Malay Peninsula | Herb | To be observed | 1 |
| Fabaceae | *Senna* | *Senna occidentalis* | North America, South America | Shrub | To be observed | 3 |
| Fabaceae | *Sesbania* | *Sesbania cannabina* | Oceania, Pacific Islands | Herb | To be observed | 51 |
| Fabaceae | *Crotalaria* | *Crotalaria pallida* | Tropical Africa | Herb | To be observed | 12 |
| Lamiaceae | *Stachytarpheta* | *Stachytarpheta jamaicensis* | Central and South America | Herb | 3 | 5 |
| Lythraceae | *Sonneratia* | *Sonneratia apetala* | Tropical Asia | Tree | To be observed | 13 |
| Malvaceae | *Malvastrum* | *Malvastrum coromandelianum* | Americas | Herb | 2 | 2 |
| Malvaceae | *Triumfetta* | *Triumfetta rhomboidea* | Africa | Herb | To be observed | 4 |
| Oxalidaceae | *Oxalis* | *Oxalis corymbosa* | South America | Herb | 4 | 16 |
| Passifloraceae | *Passiflora* | *Passiflora foetida* | West Indies and northern South America | Vine | 3 | 10 |
| Plantaginaceae | *Scoparia* | *Scoparia dulcis* | Tropical Americas | Herb | 2 | 3 |
| Plantaginaceae | *Mecardonia* | *Mecardonia procumbens* | North and South America | Herb | 4 | 2 |
| Poaceae | *Brachiaria* | *Brachiaria mutica* | West Africa and the Americas | Herb | 2 | 2 |
| Poaceae | *Panicum* | *Panicum repens* | Southern Europe | Herb | 2 | 24 |
| Poaceae | *Pennisetum* | *Pennisetum purpureum* | Africa | Herb | 3 | 1 |
| Poaceae | *Paspalum* | *Paspalum urvillei* | South America | Herb | 3 | 3 |
| Poaceae | *Panicum* | *Panicum maximum* | Tropical Africa | Herb | 3 | 1 |
| Poaceae | *Melinis* | *Melinis repens* | South Africa | Herb | 3 | 27 |
| Poaceae | *Paspalum* | *Paspalum conjugatum* | Latin America | Herb | 4 | 1 |
| Poaceae | *Cyperus* | *Cyperus surinamensis* | South America, North America, and the Caribbean | Herb | 4 | 16 |
| Poaceae | *Pennisetum* | *Pennisetum polystachion* | South America, Africa | Herb | To be observed | 8 |
| Rubiaceae | *Spermacoce* | *Spermacoce remota* | American Tropics | Herb | To be observed | 1 |
| Solanaceae | *Solanum* | *Solanum torvum* | Caribbean coast | Shrub | 1 | 13 |
| Solanaceae | *Solanum* | *Solanum americanum* | Americas | Herb | 3 | 26 |
| Solanaceae | *Physalis* | *Physalis angulata* | South America | Herb | 4 | 2 |
| Urticaceae | *Pilea* | *Pilea microphylla* | South America | Herb | 4 | 2 |
| Verbenaceae | *Lantana* | *Lantana camara* | Tropical Americas | Shrub | 1 | 47 |

TABLE S5 Summary of the 15 dominant species (7 invasive and 8 native) selected for occupancy analysis

| species | occurrence | mean_cover | total_cover | status | rel_cover_invasive | rel_cover_native |
| --- | --- | --- | --- | --- | --- | --- |
| *Bidens alba* | 0.986111 | 13.92742 | 1002.774 | Invasive | 33.997 | NA |
| *Sphagneticola trilobata* | 0.430556 | 6.174405 | 444.5572 | Invasive | 15.0718 | NA |
| *Leucaena leucocephala* | 0.666667 | 4.608353 | 331.8014 | Invasive | 11.24905 | NA |
| *Mikania micrantha* | 0.597222 | 3.960906 | 285.1853 | Invasive | 9.66862 | NA |
| *Panicum repens* | 0.291667 | 1.808707 | 130.2269 | Invasive | 4.415076 | NA |
| *Lantana camara* | 0.527778 | 1.586303 | 114.2138 | Invasive | 3.872185 | NA |
| *Sesbania cannabina* | 0.555556 | 1.511221 | 108.8079 | Invasive | 3.688909 | NA |
| *Cynodon dactylon* | 0.444444 | 3.091493 | 222.5875 | Native | NA | 6.170778 |
| *Phragmites australis* | 0.347222 | 2.487865 | 179.1263 | Native | NA | 4.965906 |
| *Saccharum arundinaceum* | 0.319444 | 2.175945 | 156.6681 | Native | NA | 4.343299 |
| *Ficus concinna* | 0.222222 | 2.092161 | 150.6356 | Native | NA | 4.176061 |
| *Acacia confusa* | 0.208333 | 2.000381 | 144.0275 | Native | NA | 3.992864 |
| *Ruellia simplex* | 0.25 | 1.955068 | 140.7649 | Native | NA | 3.902417 |
| *Apluda mutica* | 0.416667 | 1.895465 | 136.4735 | Native | NA | 3.783445 |
| *Coccinia grandis* | 0.291667 | 1.663369 | 119.7626 | Native | NA | 3.320171 |

TBALE S6 Native species richness and cover (mean ± SD) across the six habitat types

| Habitat | Native Richness (mean ± SD) | Native Cover (%) (mean ± SD) |
| --- | --- | --- |
| Forest | 34.1 ± 9.18 | 78.6 ± 8.85 |
| Forest edge | 11.2 ± 4.65 | 37.7 ± 26.2 |
| Park | 18.0 ± 4.92 | 37.3 ± 15.6 |
| Roadside | 11.5 ± 3.55 | 39.2 ± 21.3 |
| Wasteland | 18.5 ± 3.03 | 41.0 ± 13.5 |
| Wetland | 19.8 ± 9.49 | 66.3 ± 9.33 |
